# Cryptic splicing events in the iron transporter *ABCB7* and other key target genes in *SF3B1*-mutant myelodysplastic syndromes

**DOI:** 10.1038/leu.2016.149

**Published:** 2016-06-17

**Authors:** H Dolatshad, A Pellagatti, F G Liberante, M Llorian, E Repapi, V Steeples, S Roy, L Scifo, R N Armstrong, J Shaw, B H Yip, S Killick, R Kušec, S Taylor, K I Mills, K I Savage, C W J Smith, J Boultwood

**Affiliations:** 1 Bloodwise Molecular Haematology Unit, Nuffield Division of Clinical Laboratory Sciences, Radcliffe Department of Medicine, University of Oxford, Oxford, UK; 2 NIHR Biomedical Research Centre, Oxford, UK; 3 Centre for Cancer Research and Cell Biology, Queen's University Belfast, Belfast, UK; 4 Department of Biochemistry, Downing Site, University of Cambridge, Cambridge, UK; 5 The Computational Biology Research Group, Weatherall Institute of Molecular Medicine, University of Oxford, Oxford, UK; 6 Department of Haematology, Royal Bournemouth Hospital, Bournemouth, UK; 7 Dubrava University hospital and Zagreb School of Medicine, University of Zagreb, Zagreb, Croatia

## Abstract

The splicing factor *SF3B1* is the most frequently mutated gene in myelodysplastic syndromes (MDS), and is strongly associated with the presence of ring sideroblasts (RS). We have performed a systematic analysis of cryptic splicing abnormalities from RNA sequencing data on hematopoietic stem cells (HSCs) of *SF3B1*-mutant MDS cases with RS. Aberrant splicing events in many downstream target genes were identified and cryptic 3′ splice site usage was a frequent event in *SF3B1-*mutant MDS. The iron transporter *ABCB7* is a well-recognized candidate gene showing marked downregulation in MDS with RS. Our analysis unveiled aberrant *ABCB7* splicing, due to usage of an alternative 3′ splice site in MDS patient samples, giving rise to a premature termination codon in the *ABCB7* mRNA. Treatment of cultured *SF3B1*-mutant MDS erythroblasts and a CRISPR/Cas9-generated *SF3B1*-mutant cell line with the nonsense-mediated decay (NMD) inhibitor cycloheximide showed that the aberrantly spliced *ABCB7* transcript is targeted by NMD. We describe cryptic splicing events in the HSCs of *SF3B1*-mutant MDS, and our data support a model in which NMD-induced downregulation of the iron exporter *ABCB7* mRNA transcript resulting from aberrant splicing caused by mutant *SF3B1* underlies the increased mitochondrial iron accumulation found in MDS patients with RS.

## Introduction

The myelodysplastic syndromes (MDS) are a heterogeneous group of clonal hematopoietic stem cell (HSC) malignancies characterized by ineffective hematopoiesis leading to peripheral blood cytopenias, and show increasing bone marrow blasts.^1^ The MDS show frequent progression (approximately 40% of patients) to acute myeloid leukemia. Several genes involved in pre-messenger RNA splicing, including *SF3B1, U2AF1, SRSF2* and *ZRSR2*,^2, 3, 4^ have been shown to be mutated in over 50% of MDS patients, revealing a new leukemogenic pathway involving spliceosomal dysfunction.

The splicing factor *SF3B1* is the most frequently mutated gene in patients with MDS (20–28% of all cases).^5, 6^ Mutations of *SF3B1* occur in a high proportion (>80%) of MDS patients in whom the presence of ring sideroblasts (RS) is a characteristic disease feature, namely the refractory anemia with RS (RARS) and refractory cytopenia with multilineage dysplasia and RS (RCMD-RS) subtypes.^5, 7^ In the recent 2016 revision of the World Health Organization (WHO) classification for MDS, if a patient harbors an *SF3B1* mutation, a diagnosis of MDS with RS (MDS-RS) may be made if 5–14% RS are present in the bone marrow.^8^ *SF3B1* mutations are closely associated with the presence of RS, suggesting a causal relationship and making *SF3B1* the first gene showing a strong association with a particular morphological feature in MDS.^5^ RS are erythroblasts with excessive mitochondrial iron accumulation,^9^ and RARS patients with *SF3B1* mutation have altered iron distribution characterized by coarse iron deposits in comparison with RARS patients without *SF3B1* mutation.^10^ *SF3B1* mutations occur more frequently in low-risk MDS cases and are independent predictors of favorable survival in MDS.^5^ The clinical consequences of mutations in *SF3B1* are well documented in MDS, however the functional consequences of *SF3B1* mutations in human hematopoietic cells are not fully understood.

A well-recognized candidate gene for MDS with the RS phenotype is the iron transporter *ABCB7*. Our group first reported marked downregulation of *ABCB7* in MDS patients with RARS subtype.^11^ Hereditary X-linked sideroblastic anemia with ataxia is caused by partial loss-of-function mutations of *ABCB7*, which inhibit heme biosynthesis.^12^ Moreover, knockdown of ABCB7 in HeLa cells resulted in an iron-deficient phenotype with mitochondrial iron accumulation.^13^ Conditional gene targeting in mice has shown that *ABCB7* is essential for hematopoiesis.^12^

SF3B1 is a core component of the U2-small nuclear ribonucleoprotein complex and is involved in stabilizing the interaction of the U2-small nuclear ribonucleoprotein with the branch point (BP),^14^ upstream of the 3′ splice site. SF3B1 also interacts with other spliceosomal proteins such as U2AF2, which binds the polypyrimidine tract (PPT) downstream of the BP.^15, 16^ Base-pairing of U2 snRNA with the pre-messenger RNA bulges out the BP adenosine, specifying it as the site to initiate the nucleophilic attack in the first step of splicing. The binding of the SF3B complex proteins around the BP prevents the premature activity at the site before the fully active spliceosome is assembled.^17^ The role of SF3B1 and the U2-small nuclear ribonucleoprotein in recognizing and binding the BP suggest that *SF3B1* mutations may alter BP and/or 3′ splice site selection.

The splicing factor genes found to be mutated in MDS code for proteins that have a role in the recognition of 3′ splice sites during processing of pre-messenger RNAs.^3^ Altered RNA splicing has been suggested as the mechanism underlying the observed phenotypic changes concomitant to splicing factor gene mutations, including *SF3B1*, and the identification of aberrantly spliced target genes in the hematopoietic cells of *SF3B1*-mutant MDS cases is important.

A number of studies to date have used RNA sequencing (RNA-Seq) on unfractionated bone marrow mononuclear cells from a small number of *SF3B1*-mutant MDS patients.^10, 18, 19^ MDS is a disorder of the HSC and we thus studied the transcriptome of CD34^+^ cells from MDS patients with *SF3B1* mutations using RNA-Seq. We have recently identified many genes significantly differentially expressed at the transcript and/or exon level in bone marrow CD34^+^ cells of *SF3B1*-mutant MDS compared with wild-type and healthy control cases.^20^

Recently, *SF3B1* mutations have been identified in various tumor types, suggesting that somatic mutations in spliceosome genes have an important role in tumorigenesis.^21, 22, 23, 24^ *SF3B1* mutations have been shown to occur in chronic lymphocytic leukemia, uveal melanoma, breast cancer and pancreatic cancer.^24, 25^ *SF3B1* mutations have clear mutational hotspots and are considered to be gain-of-function/neomorphic mutations.^2, 3, 26, 27^ The codons most commonly affected by *SF3B1* mutations in other cancers that harbor this mutation, including chronic lymphocytic leukemia, uveal melanoma, breast cancer and pancreatic cancer, are the same as the ones affected in MDS (K700, R625 and K666). Recent studies of chronic lymphocytic leukemia, breast cancer and uveal melanoma using RNA-Seq have shown that *SF3B1* mutations are associated with differential exon usage and induce cryptic alternative 3′ splice site selection in these cancers.^28^ However, a systematic analysis of cryptic splicing abnormalities has not been performed in MDS HSCs.

In this study, we have performed an analysis of RNA-Seq data on HSCs of *SF3B1*-mutant MDS cases to identify aberrant/cryptic splicing events. The identification of the splicing aberrations induced by *SF3B1* mutation in the HSCs of MDS patients will shed light on the downstream effects that lead to the MDS phenotype and may allow for the identification of new therapeutic targets in this disease.

## Materials and methods

### Samples and RNA-Seq

RNA-Seq data were obtained from CD34^+^ cells isolated from bone marrow samples of eight MDS patients (four RARS and four RCMD-RS) with *SF3B1* mutation (four K700E, one E622D, one R625L, one H662Q and one K666R; 45–52% *SF3B1*-mutant allele expression range), four MDS cases (all refractory cytopenia with multilineage dysplasia) without mutations in the splicing factor genes *SF3B1, SRSF2, U2AF1* and *ZRSR2*, and five healthy individuals.^20^ CD34^+^ cells were isolated from bone marrow samples of the 12 MDS patients and five healthy controls using magnetic-activated cell sorting columns (Miltenyi Biotec, Bergisch Gladbach, Germany), according to the manufacturer's recommendations.

DNase-treated (Invitrogen, Carlsbad, CA, USA) total RNA was purified using XP beads (Beckman Coulter, High Wycombe, UK), and library preparation was performed using the NEBNext Ultra directional RNA Library prep kit (NEB, Hitchin, UK) following the manufacturer's recommendations. Custom indexes were used and samples were purified using XP beads (Beckman Coulter) instead of size selection. Sequencing was performed on an Illumina HiSeq2000 instrument (Illumina, San Diego, CA, USA).

### RNA-Seq data analysis

Following QC analysis with the fastQC package (http://www.bioinformatics.babraham.ac.uk/projects/fastqc), reads were aligned using STAR^29^ against the human genome assembly (NCBI build37 (hg19) UCSC transcripts). Non-uniquely mapped reads and reads that were identified as PCR duplicates using Samtools^30^ were discarded. The aligned reads were reconstructed into transcripts using Cufflinks^31^ and were then merged into a single assembly, along with known isoforms from the NCBI build37 (hg19) UCSC transcripts. This reference-guided assembly was then used as the transcripts annotation by rMATS.

Alternative 3′ and 5′ splice sites, skipped exons, mutually exclusive exons and retained introns were quantified using rMATS^32^ with the assembly produced from Cufflinks. The default parameters were used for the comparison of the samples. The results were filtered using a false discovery rate<0.05 and inclusion level difference values >0.3 or <−0.3. The alternative spliced events were then plotted using the sashimi plots of the MISO software.^33^ The results were visualized and filtered using the data visualization tool Zegami (http://zegami.com/).

### Gene ontology analysis

See Supplementary Methods.

### Analysis of 3′ splice site properties

See Supplementary Methods.

### End point RT-PCR validation of aberrant splicing isoforms

Six genes (including *ABCB7*) with cryptic 3′ splice sites were selected from the rMATS data analysis for end point reverse transcriptase-PCR (RT-PCR) validation. See Supplementary Methods for details.

### Erythroblast cell culture and cycloheximide treatment

Bone marrow CD34^+^ cells from healthy individuals were purchased from Lonza (Basel, Switzerland). Bone marrow samples were obtained and CD34^+^ cells were isolated from two MDS patients with *SF3B1* K700E mutation. CD34^+^ cells from MDS patients and from healthy controls were cultured as previously described.^34, 35^ On day 11 and day 14, an aliquot of cells were treated with 100 μg/ml cycloheximide for 4 h and subsequently collected for RNA extraction. Total RNA was reverse-transcribed using High capacity cDNA reverse transcription kit (Applied Biosystems, Foster City, CA, USA). The expression of aberrantly spliced *ABCB7* was determined by RT-PCR as described in the Supplementary Methods.

### Generation of *SF3B1*-mutant K562 cells by CRISPR/Cas9 and cycloheximide treatment

See Supplementary Methods.

### Pancreatic cell line Panc 05.04 culture and cycloheximide treatment

See Supplementary Methods.

## Results

### Cryptic splicing events in HSCs of *SF3B1*-mutant MDS

We have analyzed RNA-Seq data obtained from the CD34^+^ cells from eight MDS cases harboring *SF3B1* mutations (*SF3B1*-mutant, all with >15% RS), four MDS patients without splicing factor gene mutations (wild type) and five healthy individuals (control)^20^ using rMATS, a bioinformatics pipeline designed to detect alternative (including cryptic) splicing events involving two isoforms from an alternatively spliced region.^32^ These events are categorized as alternative 3′ splice site (A3SS) usage, alternative 5′ splice site (A5SS) usage, exon skipping, mutually exclusive exons or retained introns.

When comparing *SF3B1*-mutant to wild type, we identified 126 significant splicing events (92 genes), of which 42 were A3SS, 6 A5SS, 8 mutually exclusive exons, 13 skipped exons and 57 retained introns (Tables 1 and 2, Supplementary Table S1). When comparing *SF3B1*-mutant with controls, 213 significant splicing events (164 genes) were identified, of which 62 were A3SS, 10 A5SS, 12 mutually exclusive exon, 12 skipped exons and 117 retained intron (Tables 1 and 3, Supplementary Table S2). Top-ranking significant genes showing at least one cryptic splicing event in both of these comparisons include *TMEM14C, ENOSF1, SEPT6, DYNLL1, HINT2* and *ABCC5*.

We performed gene ontology analysis on the lists of significant genes showing aberrant splicing events identified by the rMATS pipeline using GOseq. The significant main ontology themes for the comparison of *SF3B1*-mutant with wild type and controls include ‘RNA processing' and ‘RNA splicing' (Supplementary Tables S3 and S4).

### Properties of misregulated alternative 3′ splice sites

Compared with all alternative splicing events (regulated and unregulated) detected in the data by rMATS, we found a significant overrepresentation of regulated A3SS (*P*<1 × E-08, χ^2^-test with Yates's continuity correction), but no significant overrepresentation of alternative 5′ splice sites, in the comparison of *SF3B1*-mutant with both wild type and controls (Table 1). Indeed, 15/20 (75%) most significant aberrant splicing events in the comparison of *SF3B1*-mutant with wild type (Table 2) and 13/20 (65%) most significant aberrant splicing events in the comparison of *SF3B1*-mutant with controls (Table 3) were A3SS. These data are in accord with the known function of SF3B1 in the recognition of BPs and 3′ splice sites. In addition to A3SS, retained introns were also significantly overrepresented, while cassette and mutually exclusive exons were underrepresented.

**Figure 1:**
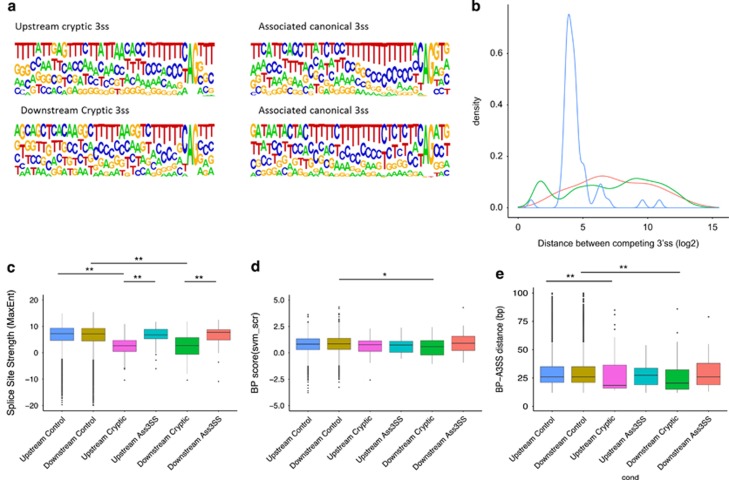
Properties of A3SS misregulated in *SF3B1*-mutant compared with wild-type *SF3B1* MDS HSCs. (**a**) Sequence logos for upstream and downstream cryptic 3′ splice sites along with their associated canonical sites. (**b**) Density plot showing distance (log2) between pairs of 3′ splice sites. Blue line: upstream cryptic. Red line: downstream cryptic. Green line: A3SS unaffected by *SF3B1* mutation. (**c**) 3′ splice site strengths (maximum entropy) for upstream and downstream control (unregulated) A3SS, upstream cryptic sites and their associated canonical sites, and downstream cryptic sites and their associated canonical sites. (**d**) BP scores. (**e**) Distance of highest scoring predicted BP from associated 3′ splice site. (*P<0.05, **P<0.01).

The majority of the regulated A3SS events involved use of an A3SS upstream of the canonical 3′ splice site (Table 1). Analysis of the sequences of upstream and downstream cryptic 3′ splice sites, along with their associated canonical 3′ splice sites revealed distinct sequence features. Both sets of canonical 3′ splice sites had extensive 18–20 nt PPTs with enrichment of uridines at most positions (Figure 1a, Supplementary Figure S1A). In contrast, the upstream cryptic sites had a shorter PPT of ~8 nt with a stretch of 4–5A residues 13–17 nt upstream. Downstream cryptic sites in the comparison of *SF3B1*-mutant with wild type also had more purine interruptions (Figure 1a), but this was not evident in the comparison of *SF3B1*-mutant with control (Supplementary Figure S1A). Analysis of the distance separating pairs of A3SS revealed a very distinct pattern for upstream cryptic 3′ splice sites, with a strong peak at 15nt (Figure 1b, Supplementary Figure S1B, blue trace; 15nt=3.9 on log2 scale). In contrast, downstream cryptic 3′ splice sites and A3SS unregulated by the *SF3B1* mutation showed a much broader distribution of spacing with most pairs of A3SS being much more widely spaced (red and green traces in Figure 1b and Supplementary Figure S1B). One interesting exception was a peak at 3 nt (1.6 on log2 scale) for the unregulated A3SS, which corresponds to the so-called NAGNAG class of A3SS,^36^ selection of which occurs at step 2 of splicing^37^ consistent with a lack of effect of *SF3B1* mutation. Comparison of 3′ splice site strength showed that both upstream and downstream cryptic 3′ splice sites were weaker than their associated canonical sites in the comparison of *SF3B1*-mutant with wild type (Figure 1c), but in the comparison of *SF3B1*-mutant with controls, only the upstream cryptic sites were weaker (Supplementary Figure S1C). This is consistent with the adenosine interruptions of the PPT in the upstream cryptic sites (Figure 1a, Supplementary Figure S1A). We used SVM-BP finder^38^ to predict the location and strength of the top-scoring predicted BPs associated with each A3SS. Although the predicted BP scores did not differ significantly between sets of regulated cryptic 3′ splice site and their associated canonical sites (Figure 1d, Supplementary Figure S1D), top-scoring BPs for upstream cryptic sites were closer (median 18nt, BP-AG distance) than those for their associated canonical sites (median 27nt) or unregulated A3SS (median 26nt) (Figure 1e, Supplementary Figure S1E). Taken together, our data show that MDS-associated *SF3B1* mutations result in widespread use of cryptic 3′ splice sites a short distance upstream of canonical sites and could be consistent either with use of a common BP in association with upstream cryptic and canonical splice site pairs^28^ or, more likely, with shifted use of both BP and consequently 3′ splice site in the *SF3B1*-mutant,^39, 40^ as shown in recent reports in other cancers.

### Validation of cryptic 3′ splice site events

**Figure 2:**
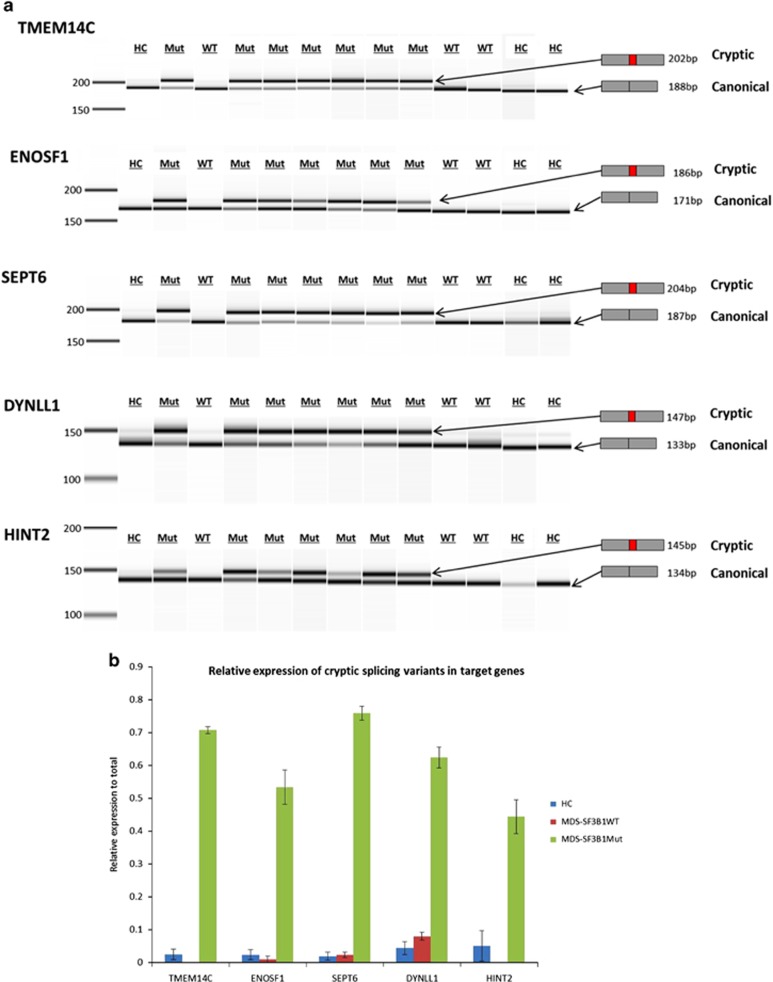
Validation and semi-quantification of cryptic 3′ splice site usage in five selected genes from the rMATS analysis in *SF3B1*-mutant MDS HSC samples. (**a**) PCR products of the five genes (*TMEM14C, ENOSF1, DYNLL1, SEPT6* and *HINT2*) were amplified from *SF3B1*-mutant MDS samples (Mut), wild-type MDS samples (WT) and healthy control samples (HC) run on an Agilent 2100 Bioanalyzer instrument using the DNA 1000 kit. The aberrant transcripts (indicated by the higher band) were observed in the *SF3B1*-mutant MDS samples. (**b**) Semi-quantification of the PCR bands showed high cryptic to canonical isoform ratio in the *SF3B1*-mutant MDS samples.

Several of the misregulated A3SS events identified by the rMATS pipeline were validated using RT-PCR in patient and healthy control samples. We chose *TMEM14C, SEPT6, HINT2, DYNLL1* and *ENOSF1* for validation. These genes all showed upstream cryptic 3′ splice sites in the rMATS analysis with the cryptic AG site located between 11 and 17 nucleotides upstream of the canonical site (Supplementary Figure S2). Aberrant splicing of *TMEM14C* and *DYNLL1* introduces an addition of 14 base pairs in the 5′UTR region of these genes. The cryptic splice site event in *ENOSF1* leads to the addition of 15 base pairs (encoding five amino acids) to the coding sequence. Aberrant splicing of *SEPT6* and *HINT2* introduces an addition of 17 and 11 base pairs, respectively, leading to a frameshift in *SEPT6* and to the rise of a premature termination codon in the first 3 base pairs of exon 5 in *HINT2*. The RT-PCR results confirmed the cryptic splicing events identified by RNA-Seq in these five genes in MDS patient samples with *SF3B1* mutation (Figures 2a and b).

### Cryptic splicing of *ABCB7* in HSCs of *SF3B1*-mutant MDS

**Figure 3:**
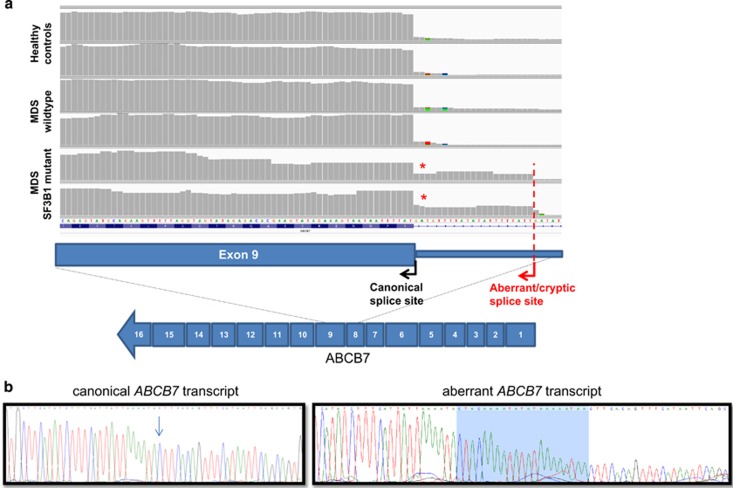
Cryptic 3′ splice site usage of *ABCB7* in *SF3B1-*mutant MDS HSCs. (**a**) Visualization of RNA-Seq traces for the *ABCB7* gene (intron 8-exon 9 junction) in two healthy controls, two MDS patients with no known splicing factor mutations and two *SF3B1-*mutant MDS cases, using Integrative Genomics Viewer (IGV). A cryptic 3′ splice site is observed in the *ABCB7* gene between exon 8 and exon 9 in the *SF3B1*-mutant MDS cases, leading to an addition of 21 base pairs to the coding sequence causing a premature termination codon at the seventh amino acid (indicated with *). (**b**) Sanger sequencing traces of gel extracted bands corresponding to the canonical *ABCB7* transcript and to the aberrant *ABCB7* transcript containing the additional 21 base pairs (highlighted in blue).

**Figure 4:**
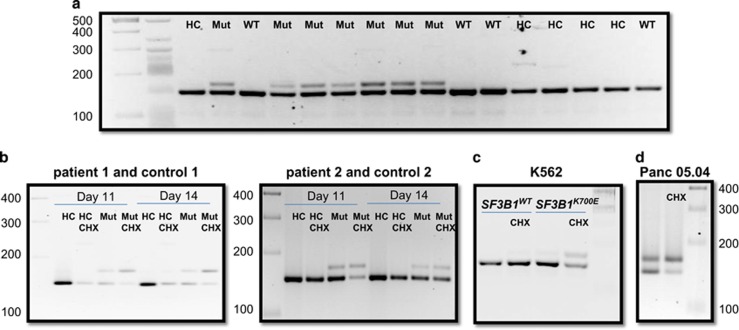
Aberrant splicing of *ABCB7* in HSCs and cultured erythroblast from *SF3B1-*mutant MDS samples, in the K562-SF3B1^K700E^ cell line and a *SF3B1*-mutant pancreatic cell line. (**a**) RT-PCR confirmation of aberrant splicing of *ABCB7* in *SF3B1-*mutant MDS HSC samples. The higher 174 bp band corresponds to the aberrant *ABCB7* transcript and the lower 153 bp band corresponds to the canonical *ABCB7* transcript. The aberrant *ABCB7* transcript (higher 174 bp band) was observed in the *SF3B1*-mutant MDS samples (Mut), but not in samples from wild-type MDS patients (WT) or in samples from healthy controls (HC). (**b**) Aberrant splicing of *ABCB7* in cultured erythroblast from *SF3B1*-mutant MDS patients. Each panel shows data from one different *SF3B1*-mutant MDS patient (Mut) and from one different healthy control (HC). RT-PCR for *ABCB7* was performed on cultured erythroblasts at day 11 and day 14 of culture treated with and without the NMD inhibitor cycloheximide (CHX). The higher 174 bp band corresponds to the aberrant *ABCB7* transcript and the lower 153 bp band corresponds to the canonical *ABCB7* transcript. The RT-PCR results showed an increase in the product corresponding to the aberrant *ABCB7* transcript (higher 174 bp band) in the *SF3B1*-mutant patient samples treated with CHX compared with untreated samples. No aberrant *ABCB7* splicing was seen in the samples from healthy controls. (**c**) Aberrant splicing of *ABCB7* in K562-SF3B1^K700E^. RT-PCR for *ABCB7* was performed on cultured K562-SF3B1^K700E^ and K562-SF3B1^WT^ cells treated with and without the NMD inhibitor CHX. The higher 174 bp band corresponds to the aberrant *ABCB7* transcript and the lower 153 bp band corresponds to the canonical *ABCB7* transcript. The RT-PCR results showed an increase in the product corresponding to the aberrant *ABCB7* transcript (higher 174 bp band) in the K562-SF3B1^K700E^ cells treated with cycloheximide (CHX) compared with the untreated cells. No aberrant *ABCB7* splicing was seen in the K562-SF3B1^WT^ cells untreated or treated with CHX. (**d**) Aberrant splicing of *ABCB7* in Panc 05.04 cells with *SF3B1* mutation (K700E). RT-PCR for *ABCB7* was performed on cultured Panc 05.04 cells treated with and without the NMD inhibitor CHX. The higher 174 bp band corresponds to the aberrant *ABCB7* transcript and the lower 153 bp band corresponds to the canonical *ABCB7* transcript. The RT-PCR results showed an increase in the product corresponding to the aberrant *ABCB7* transcript (higher 174 bp band) in the Panc 05.04 cells treated with CHX compared with the untreated cells.

We previously reported marked downregulation of the iron transporter *ABCB7* in MDS patients with RS.^11^ In this study, we identified a significant A3SS event (false discovery rate=0.006, inclusion level difference=−0.184) in the *ABCB7* gene in the comparison of *SF3B1*-mutant with controls. Aberrant splicing introduces an addition of 21 base pairs from the intronic region between exons 8 and 9 causing an addition of seven amino acids to the protein sequence with the last 3 base pairs proximal to the canonical exon 9 giving rise to a premature termination codon (Figures 3a and b). This result was confirmed by RT-PCR in patient and healthy control HSC samples: the aberrant *ABCB7* transcript was observed in all *SF3B1*-mutant MDS samples analyzed (*n*=7), but not in any of the samples from wild-type patients (*n*=4) or from healthy controls (*n*=5) (Figure 4a). Sanger sequencing of gel extract bands confirmed the presence of the addition of 21 base pairs in the aberrant *ABCB7* transcript (Figure 3b). These data demonstrate that aberrant splicing of *ABCB7* observed is specific to MDS cases carrying mutation of *SF3B1*.

### NMD targets the aberrantly spliced ABCB7 transcript in *SF3B1*-mutant MDS erythroblasts, isogenic K562-SF3B1^K700E^ and a *SF3B1*-mutant pancreatic cell line

The presence of premature termination codons can lead to degradation of mRNA transcripts by nonsense-mediated RNA decay (NMD).^41^ We thus investigated whether the aberrantly spliced *ABCB7* transcript containing a premature termination codon that we identified in *SF3B1*-mutant MDS is affected by NMD. CD34^+^ cells from two *SF3B1*-mutant MDS RARS patients and from two healthy controls were cultured using a method developed to study the generation of erythroblasts.^34, 35^ Cells were collected at day 11 and day 14 of erythroid culture and treated with cycloheximide (an inhibitor of protein biosynthesis known to impair NMD^42^), and subjected to RT-PCR for *ABCB7*. The RT-PCR results showed an increase in the product corresponding to the aberrant *ABCB7* transcript in the *SF3B1*-mutant patient samples treated with cycloheximide compared with untreated samples (Figure 4b), indicating that NMD targets the aberrantly spliced *ABCB7* transcript and underlies the downregulation of *ABCB7* observed in *SF3B1*-mutant MDS patients.

In addition, we performed RT-PCR for *ABCB7* in K562-SF3B1^K700E^ and SF3B1^WT^ isogenic cells obtained using CRISPR/Cas9 gene editing and the pancreatic cell line Panc 05.04, which has a heterozygous SF3B1 K700E mutation, and we observed the same *ABCB7* cryptic 3′ splice site event (Figures 4c and d), which introduces a stop codon as identified in the CD34^+^ cells and cultured erythroblasts of *SF3B1*-mutant MDS patients. Treatment of K562-SF3B1^K700E^ and Panc 05.04 cells with cycloheximide resulted in an increase of the aberrantly spliced form of the *ABCB7* transcript (Figures 4c and d).

## Discussion

The splicing factor *SF3B1* is the most frequently mutated gene found in MDS, and is strongly associated with the RS phenotype.^5, 6^ It is still unknown how *SF3B1* mutations lead to the formation of RS in MDS. Given the critical functions of SF3B1 on 3′ splice site recognition, the probable consequence of this spliceosome mutation is aberrant splicing of various downstream target genes in MDS. The MDS arise in the HSC population in the bone marrow and *SF3B1* is a founder mutation.^43, 44^ It is important to study the impact of *SF3B1* mutation on the transcriptome in the cell of origin. We sought to identify the aberrant/cryptic mRNA splicing events associated with the *SF3B1* mutation in the HSCs of MDS patients. We performed an analysis of RNA-Seq data that we generated from CD34^+^ cells isolated from bone marrow samples of SF3B1-mutant MDS patients with RS, MDS patients with no splicing factor gene mutation and from healthy individuals.^20^

The recent identification of A3SS usage in other malignancies with *SF3B1* mutation^22, 28^ highlights the importance of interrogating RNA-seq data using a method that allows the identification of not only annotated alternative splicing events, but also of *de novo* cryptic splicing events. In this study, we used *de novo* transcriptome reconstruction and the rMATS pipeline to identify unannotated alternative splicing events, including A3SS and retained introns. We have found that *SF3B1* mutations are associated with various aberrant splicing events in the HSCs of MDS patients. Interestingly, we identified significant cryptic 3′ splice site usage affecting many genes when comparing the transcriptome of *SF3B1*-mutant MDS cases with that of MDS wild-type and control cases, in accord with the known role of SF3B1 in the recognition of 3′ splice sites. A3SS events were significantly overrepresented in both comparisons, but no significant overrepresentation of alternative 5′ splice sites was observed. Indeed, the majority (65–75%) of the most significant aberrant splicing events in the comparison of *SF3B1*-mutant with both wild type and controls were A3SS. Furthermore, the number of genes with A3SS was sixfold to sevenfold higher than the number of genes with alternative 5′ splice sites. These data show that aberrant 3′ splice site selection is a frequent and important event associated with *SF3B1* mutations in the HSCs of MDS patients.

The most distinct group of misregulated events in *SF3B1-*mutant MDS involved the use of cryptic 3′ splice sites located 15–20 nt upstream of the canonical 3′ splice site, consistent with recent reports in other cancers.^28, 39, 40^ This is an unusual location as the typical 3′ splice site arrangement comprises a BP typically 18–40 nt upstream of the 3′ splice site, with an optimal separation of 19–23 nt^45^ and a minimal separation of ~12 nt.^37^ The 15 nt separation of A3SS could therefore be consistent with a single BP-PPT unit from which either of two 3′ splice sites can be used,^28^ similar to *Drosophila* SXL exon 3.^46^ Alternatively, *SF3B1* mutations could lead to a shift in BP selection a short distance upstream leading to altered 3′ splice site selection.^39, 40^ For the six events that we investigated, the predicted and mapped BP locations support the second scenario in which altered BP selection drives the change in 3′ splice site selection in MDS (Supplementary Figure S2). This would be consistent with the recruitment of U2-small nuclear ribonucleoprotein to positions 5′ of the BP in response to SF3B1 targeting drugs.^47^ Emerging data suggest that myeloid malignancies with splicing factor gene mutations are preferentially susceptible to additional splicing perturbations induced by splicing factor inhibitors^48^ and this may also represent a therapeutic approach in *SF3B1*-mutant MDS.

In our study, we found at least one cryptic splicing event in *TMEM14C, ENOSF1, DYNLL1, SEPT6* and *HINT2* (validated by RT-PCR) when comparing *SF3B1*-mutant to wild type and to controls. Importantly, cryptic splicing events affecting *TMEM14C, ENOSF1, DYNLL1* and *HINT2* have been associated with *SF3B1* mutation in other cancers such as chronic lymphocytic leukemia, uveal melanoma and breast cancer.^21, 22, 23^ Emerging evidence thus suggests that there are several common downstream target genes in *SF3B1*-mutant malignancies, which may have implications for the design of new therapies for this group of cancers.

It is most probable that several target genes showing cryptic splicing contribute to the phenotype observed in MDS patients with the *SF3B1* mutation. We note for example that TMEM14C has an important role in the terminal steps of the heme synthesis pathway.^49^

MDS patients with RARS suffer from a refractory anemia and show erythroid hyperplasia and ineffective erythropoiesis as a result of increased apoptosis in the bone marrow.^50^ RS are characterized by excessive iron accumulation in the mitochondria of erythroid progenitors.^9^ The close association between *SF3B1* mutations and RS is consistent with a causal relationship, and makes this the first gene to be strongly associated with a specific feature of MDS.^5^ How *SF3B1* mutations affect formation of RS is still unknown.

Cryptic splicing of genes involved in iron homeostasis and/or hemoglobin synthesis could have a role in the ineffective erythropoiesis observed in MDS patients with *SF3B1* mutation and RS.^51^ We reported some years ago a strong relationship between an increasing percentage of bone marrow RS in MDS patients and decreasing expression levels of the iron transporter *ABCB7*.^11^ The *ABCB7* gene is the functional ortholog of the yeast Atm1p gene,^52^ which has been shown to be required for mitochondrial iron homeostasis^53^ and is involved in the transport of a component required for the maturation of iron-sulfur cluster proteins from the mitochondria to the cytosol.^12^ Functional studies showing that forced expression of *ABCB7* can restore erythroid growth and survival of RARS progenitors while decreasing the expression of aberrant mitochondrial ferritin (a marker for aberrant iron accumulation) subsequently implicated ABCB7 in the phenotype of acquired sideroblastic anemia (RARS).^54^ In a recent study, we performed an integrative analysis in MDS and the strongest association found was between the presence of *SF3B1* mutations and marked downregulation of *ABCB7*.^55^ Given the strong correlation between *SF3B1* mutations and the presence of RS,^5^ our data suggested a three-way association among *SF3B1* mutation, *ABCB7* downregulation and the occurrence of RS. Therefore, the marked downregulation of *ABCB7* observed in MDS patients with RARS has been recognized as an important finding for several years; however, the mechanism underlying the downregulation of this gene in MDS has remained a mystery.

In the current study, the use of a pipeline that can detect cryptic splicing events has enabled the identification of A3SS usage of *ABCB7* in the HSC of *SF3B1*-mutant MDS patients. Importantly, this event leads to aberrant splicing of the *ABCB7* mRNA transcript, resulting in the addition of seven amino acids, including a premature termination codon, to the protein sequence in patient samples. It is recognized that the presence of premature termination codons can lead to degradation of the mRNA transcript by NMD,^41^ and we hypothesized that this event underlies the marked downregulation of *ABCB7* observed in the HSCs of *SF3B1*-mutant MDS patients. This mechanism is strongly supported by our data on cultured *SF3B1*-mutant MDS erythroblasts treated with the NMD inhibitor cycloheximide, showing that the aberrantly spliced *ABCB7* transcript is targeted by NMD in erythroid cells. Importantly, we next showed that the aberrantly spliced *ABCB7* transcript was present in the myeloid cell line K562 in which the *SF3B1* mutation was introduced using CRISPR/Cas9 gene editing, and in the pancreatic cell line Panc 05.04 (which is mutant for *SF3B1*). Treatment of these two cell lines with cycloheximide resulted in an increase of the aberrantly spliced form of the *ABCB7* transcript. These data provide strong evidence that the *SF3B1* mutation leads to aberrant *ABCB7* splicing and downregulation via NMD in human myeloid cells and other cancer cells. Aberrant splicing of *ABCB7* has been reported recently in an isogenic *SF3B1*-mutant pre-B ALL cell line in another study.^39^ We suggest that downregulation of the iron exporter *ABCB7* resulting from aberrant splicing of the mRNA transcript leading to NMD underlies the increased mitochondrial iron accumulation found in MDS patients with RS. Our data provide an important link between inherited and acquired forms of sideroblastic anemia.

It is possible that the detection of aberrantly spliced target genes, in particular *ABCB7*, by RT-PCR could form the basis of a new diagnostic test for *SF3B1*-mutated MDS and may provide valuable information in cases with suspected MDS.

Our study is the first to describe the cryptic splicing events that occur in the hematopoietic progenitor cells of *SF3B1*-mutant MDS. These data illuminate the downstream target genes that may have a role in the development of the MDS phenotype, and further our understanding of the effect of *SF3B1* mutations on splicing in malignancy. We demonstrate a mechanism linking the presence of *SF3B1* mutation in MDS RARS patients and the NMD-induced marked downregulation of the iron transporter *ABCB7*, and provide strong evidence supporting a critical role of ABCB7 in the development of the RS phenotype. ABCB7 might represent a therapeutic target in MDS with RS.

## Figures and Tables

**Table 1:** Number of significant cryptic splicing events in the comparison of *SF3B1*-mutant MDS cases with wild-type MDS cases and with healthy controls, and breakdown by event type

	*Mutant vs wild type*	P*-value*	*Direction of change*	*wt-mut*	*Mutant vs Control*	P*-value*	*Direction of change*	*HC - mut*
No. of significant events (genes)	126 (92)			+/−	213 (164)			+/−
A3SS	42	<1e-8	Overrepresented	21/21	62	<1e-8	Overrepresented	25/37
A5SS	6	0.236	Not significant	6/0	10	0.38	Not significant	9/1
MXE	8	0.00002	Underrepresented	2/6	12	0.0047	Underrepresented	2/10
RI	57	<1e-8	Overrepresented	55/2	117	<1e-8	Overrepresented	115/2
SE	13	<1e-8	Underrepresented	5/8	12	<1e-8	Underrepresented	3/9

Abbreviations: A3SS, alternative 3′ splice site usage; A5SS, alternative 5′ splice site usage; MXE, mutually exclusive exons; RI, retained introns; SE, exon skipping.

The *P*-value was obtained by Chi-squared test with Yates' correction comparing the proportion of significant events of each type to the proportion of total events of that type.

**Table 2:** List of the 20 most significant cryptic splicing events in the comparison of *SF3B1*-mutant MDS cases with wild-type MDS cases

*GeneID*	*geneSymbol*	*event_type*	*event_class*	*chr*	*strand*	*start_loc*	*end_loc*	*IncLevelDifference*	P*-value*	*FDR*
XLOC_028687	ORAI2	A3SS	Both	chr7	+	102073977	102076780	−0.732	0	0
XLOC_005007	CCDC88B	A3SS	Both	chr11	+	64119602	64120359	−0.647	0	0
XLOC_025420	TMEM14C	A3SS	Both	chr6	+	10723148	10724866	−0.623	0	0
XLOC_024609	ZDHHC11	SE	Both	chr5	−	865529	870690	−0.558	0	0
XLOC_008832	DLST	A3SS	Both	chr14	+	75355978	75356655	−0.552	0	0
XLOC_014206	ENOSF1	A3SS	Both	chr18	−	683246	686008	−0.538	0	0
XLOC_033494	SEPT6	A3SS	Both	chrX	−	118759298	118763471	−0.519	0	0
XLOC_026718	MICAL1	A3SS	Both	chr6	−	109766974	109767615	−0.517	0	0
XLOC_006947	DYNLL1	A3SS	Both	chr12	+	120933859	120934356	−0.485	0	0
XLOC_012879	MYO15B	A3SS	Both	chr17	+	73587253	73587793	−0.471	0	0
XLOC_031822	HINT2	A3SS	Both	chr9	−	35812957	35813335	−0.408	0	0
XLOC_012879	MYO15B	SE	Both	chr17	+	73597519	73598675	−0.394	0	0
XLOC_002049	SERBP1	A3SS	Both	chr1	−	67890571	67890906	−0.336	0	0
XLOC_012590	COASY	RI	Both	chr17	+	40713975	40715340	0.44	0	0
XLOC_028659	PILRB	A3SS	Both	chr7	+	99954373	99955989	0.488	0	0
XLOC_024347	ANKHD1-EIF4EBP3	A3SS	Both	chr5	+	139815689	139818202	0.598	2.55E-15	1.77E-12
XLOC_021918	UBA7	RI	Both	chr3	−	49848417	49848888	0.444	6.66E-16	2.15E-12
XLOC_012879	MYO15B	RI	Both	chr17	+	73583687	73587793	0.307	2.51E-14	5.41E-11
XLOC_026672	MAP3K7	A3SS	Both	chr6	−	91269795	91271386	-0.649	2.37E-13	1.44E-10
XLOC_028659	PILRB	A3SS	Both	chr7	+	99943488	99947510	-0.441	2.84E-13	1.65E-10

Abbreviations: A3SS, alternative 3′ splice site usage; A5SS, alternative 5′ splice site usage; FDR, false discovery rate; RI, retained introns; SE, exon skipping. The '−' sign in the IncLevelDifference corresponds to the longer isoform (exon inclusion, retained intron, upstream A3SS, downstream A5SS) being produced in *SF3B1*-mutant cells. Conversely, the '+' sign in the IncLevelDifference corresponds to upregulation of the longer isoform in wild-type MDS cases.

**Table 3:** List of the 20 most significant cryptic splicing events in the comparison of SF3B1-mutant MDS cases with healthy controls

*GeneID*	*geneSymbol*	*event_type*	*event_class*	*chr*	*strand*	*start_loc*	*end_loc*	*IncLevelDifference*	P*-value*	*FDR*
XLOC_028687	ORAI2	A3SS	Both	chr7	+	102073977	102076780	-0.726	0	0
XLOC_025420	TMEM14C	A3SS	Both	chr6	+	10723148	10724866	-0.652	0	0
XLOC_026672	MAP3K7	A3SS	Both	chr6	−	91269795	91271386	-0.641	0	0
XLOC_024609	ZDHHC11	SE	Both	chr5	−	865529	870690	-0.586	0	0
XLOC_008832	DLST	A3SS	Both	chr14	+	75355978	75356655	-0.546	0	0
XLOC_014206	ENOSF1	A3SS	Both	chr18	−	683246	686008	-0.523	0	0
XLOC_033494	SEPT6	A3SS	Both	chrX	−	118759298	118763471	-0.52	0	0
XLOC_026718	MICAL1	A3SS	Both	chr6	−	109766974	109767615	-0.509	0	0
XLOC_010867	CHTF18	A3SS	Both	chr16	+	843144	844201	-0.498	0	0
XLOC_006947	DYNLL1	A3SS	Both	chr12	+	120933859	120934356	-0.482	0	0
XLOC_012879	MYO15B	A3SS	Both	chr17	+	73587253	73587793	-0.457	0	0
XLOC_012879	MYO15B	SE	Both	chr17	+	73597519	73598675	-0.379	0	0
XLOC_015516	SAFB2	MXE	Both	chr19	−	5592757	5598895	-0.365	0	0
XLOC_031822	HINT2	A3SS	Both	chr9	−	35812957	35813335	-0.348	0	0
XLOC_002049	SERBP1	A3SS	Both	chr1	−	67890571	67890906	-0.336	0	0
XLOC_016489	TMEM214	MXE	Both	chr2	+	27260429	27261683	-0.317	0	0
XLOC_025420	TMEM14C	MXE	Both	chr6	+	10723148	10726241	-0.305	0	0
XLOC_025700	ADCY10P1	A3SS	Both	chr6	+	41040701	41046903	-0.3	0	0
XLOC_012879	MYO15B	RI	Both	chr17	+	73583687	73587793	0.356	0	0
XLOC_020345	GTSE1	RI	Both	chr22	+	46722333	46725464	0.363	0	0

Abbreviations: A3SS, alternative 3′ splice site usage; A5SS, alternative 5′ splice site usage; FDR, false discovery rate; MXE, mutually exclusive exons; RI, retained introns; SE, exon skipping. The '−' sign in the IncLevelDifference corresponds to the longer isoform (exon inclusion, retained intron, upstream A3SS, downstream A5SS) being produced in *SF3B1*-mutant cells. Conversely, the '+' sign in the IncLevelDifference corresponds to upregulation of the longer isoform in the healthy controls.
