# Supplementary material for: Cryptic splicing events in the iron transporter ABCB7 and other key target genes in SF3B1-mutant myelodysplastic syndromes
Source: Leukemia. 2016 Jun 17;30(12):2322–31. doi: 10.1038/leu.2016.149 (PMC5029572; doi:10.1038/leu.2016.149)
Supplement: Supplementary Information [file leu2016149x1.doc]

**Supplementary Information**

**Cryptic splicing events in the iron transporter *ABCB7* and other key target genes in *SF3B1* mutant myelodysplastic syndromes**

Hamid Dolatshad^1^*, Andrea Pellagatti^1^*, Fabio G. Liberante^2^, Miriam Llorian^3^, Emmanouela Repapi^4^, Violetta Steeples^1^, Swagata Roy^1^, Laura Scifo^1^, Richard N. Armstrong^1^, Jacqueline Shaw^1^, Bon Ham Yip^1^, Sally Killick^5^, Rajko Kušec^6^, Stephen Taylor^4^, Ken I. Mills^2^, Kienan I. Savage^2^, Chris W. J. Smith^3^, Jacqueline Boultwood^1^

**This file contains all legends for the Supplementary Figures and Tables, as well as the Supplementary Methods.**

**Supplementary Figure 1. Properties of A3SS misregulated in *SF3B1*-mutant MDS compared to healthy control HSCs.** (A) Sequence logos for upstream and downstream cryptic 3’ splice sites along with their associated canonical sites. (B) Density plot showing distance (log2) between pairs of 3’ splice sites. Blue line: upstream cryptic. Red line: downstream cryptic. Green line: A3SS unaffected by *SF3B1* mutation. (C) 3’ splice site strengths (Maximum Entropy) for upstream and downstream control (unregulated) A3SS, upstream cryptic sites and their associated canonical sites, and downstream cryptic sites and their associated canonical sites. (D) Branch point score. (E) Distance of highest scoring predicted BP from associated 3’ splice site. (* P<0.05, ** P<0.01)

**Supplementary Figure 2.** Arrangement of splice site and branch point elements of A3SS events in Figures 2, 3.

**Supplementary Figure 3.** Chromatogram of K562-SF3B1^WT^ and K562-SF3B1^K700E^ cell DNA illustrating the A>G (K700E, black arrow) and T>C (synonymous substitution, V701, Blue arrow) transitions successfully introduced.

**Supplementary Table 1.** Full list of significant cryptic splicing events (FDR<0.05 and IncLevelDifference >0.3 or <-0.3) in the comparison of *SF3B1*-mutant MDS cases to wildtype MDS cases. Genes are ranked by FDR.

**Supplementary Table 2.** Full list of significant cryptic splicing events (FDR<0.05 and IncLevelDifference >0.3 or <-0.3) in the comparison of *SF3B1*-mutant MDS cases to healthy controls. Genes are ranked by FDR.

**Supplementary Table 3.** Significant ontology themes identified by gene ontology analysis (GOseq) of the significant genes (FDR<0.05) showing cryptic splicing in the comparison of *SF3B1*-mutant MDS cases to wildtype MDS cases. Ontology themes within the biological process (BP) and the molecular function (MF) gene ontology domains are shown.

**Supplementary Table 4.** Significant ontology themes identified by gene ontology analysis (GOseq) of the significant genes (FDR<0.05) showing cryptic splicing in the comparison of *SF3B1*-mutant MDS cases to healthy controls. Ontology themes within the biological process (BP) and the molecular function (MF) gene ontology domains are shown.

**Supplementary Table 5.** PCR conditions for the validation of cryptic splicing events in selected genes identified by rMATS.

**Supplementary Methods**

*Samples*

MDS patient bone marrow samples were obtained with written informed consent and ethics committee approval.

*Gene Ontology analysis*

Gene Ontology analysis of the RNA-Seq data was performed using GOseq.[^1^](#_ENREF_1) A weighted bias correction based on the number of exons in each gene from the NCBI build37 (hg19) UCSC transcripts was applied.

*Analysis of 3’ss properties*

For analysis of 3’ss properties, we collected data sets from the A3SS rMATS output on the basis of FDR <0.05. For events with IncLevelDifference < -0.15 the upstream A3SS was classified as cryptic and the downstream A3SS as associated canonical A3SS. Likewise, for events IncLevelDifference > 0.15, the downstream A3SS was classified as cryptic A3SS and the upstream one associated canonical. We obtained 61 upstream A3SS and 40 downstream A3SS for the SF3B1 mutant vs wildtype dataset, and 85 upstream A3SS and 70 downstream for the SF3B1 mutant vs healthy controls.

Human sequences around A3SS and upstream introns were retrieved from UCSC (hg19, Feb. 2009) using R (http://www.R-project.org.) and the Bioconductor packages, Genomic Ranges,[^2^](#_ENREF_2) Genomic Features, biomaRt and BSgenome.Hsapiens.UCSC.hg19. Graphical outputs were generated with the CRAN package ggplot2. Statistical analysis comparing sequences properties between data sets were done using Two sided Mann-Whitney test in R.

Sequence logos were produced using WebLogo (http://weblogo.berkeley.edu/logo.cgi). For that 35nt upstream and 3 nt downstream were extracted for each A3SS as mentioned above, and fed into the web tool.[^5^](#_ENREF_5)

Density plots: For each pair of A3SS, the distance between was calculated as the difference of its chromosomal coordinates, and the log2 of the difference was plot using density plot in ggplot2.[^4^](#_ENREF_4)

Splice site strength was calculated using MaxEntScan::Score3ss. (http://genes.mit.edu/burgelab/maxent/Xmaxentscan_scoreseq_acc.html). Following developer’s instructions 23nt upstream of A3SS and 3 nt downstream were extracted as indicated above. MaxEnt scores were plotted using ggplot2.[^6^](#_ENREF_6)

Branch point scores and distance between branch point and 3’ss was obtained using SVM-BPfinder (http://regulatorygenomics.upf.edu/Software/SVM_BP/). The svm_getfeat.py script was modified to allow for BP to be 12 bp from the 3’ss by setting minidist3ss=12. Data was then filtered by restricting the predicted BP to be located between the 3’ss and 20 bp upstream of the agez, and selecting the top svm_scr scoring for each event.[^7^](#_ENREF_7)

*End point RT-PCR validation of aberrant splicing isoforms*

Total RNA isolated from the CD34^+^ cells of MDS patients and controls was reverse transcribed using High capacity cDNA reverse transcription kit (Applied Biosystems, Foster City, CA, USA). Primers (Supplementary Table 5) were designed to obtain an amplification product that spans the Cufflinks-predicted spliced-exon junctions. The PCR reactions were carried out as described in Supplementary Table 5, cleaned up using the Qiaquick PCR cleanup kit (Qiagen). The Agilent 2100 Bioanalyzer was used to visualize the aberrantly spliced isoforms in relation to the canonical isoform. For the *ABCB7* gene, PCR reactions were carried out using primers/conditions described in Supplementary Table 6, and PCR products were run on 4% agarose gel and visualized using a UV transilluminator.

*Generation of SF3B1 mutant K562 cells by CRISPR/Cas9 and cycloheximide treatment*

A truncated[^8^](#_ENREF_8) 19bp gRNA (GTTCGGACCATCAGTGCTT) targeting the K700E genomic locus was cloned in to the pSpCas9(BB)-2A-GFP (pX458) (Addgene #48138) plasmid, which expresses Cas9, and verified by sequencing.

In order to introduce the K700E mutation a single-stranded DNA oligonucleotide (ssODN) was designed and purchased as an Ultramer™ from IDT. In addition to the A>G transition at the K700E locus, a synonymous T>C transition mutation was introduced in the V701 codon to create a new restriction site. Importantly, this synonymous substitution was chosen as it was predicted – by Human Splicing Finder 3.0[^9^](#_ENREF_9) – not to affect splicing of this exon; the target region being close to an intron-exon boundary. The ssODN was homologous to 77bp either side of the introduced mutations.

The Cas9/gRNA plasmid and ssODN were nucleofected into K562 cells using the Amaxa™ 4D-Nucleofector™. After 48 hours of recovery in complete medium they were sorted by FACS (BD FACSAria II) for GFP+. Positive cells were then further incubated for another 48 hours before dilutional-cloning into 384-well plates and screening by PCR and restriction fragment length polymorphism (RFLP) using MspI (Thermo Scientific). Positive clones were confirmed by Sanger sequencing of the locus (FigureS3).

Cells were treated with 100µg/ml cycloheximide for 4 hours and collected for RNA extraction. Total RNA was reverse transcribed using High capacity cDNA reverse transcription kit (Applied Biosystems). The expression of aberrantly spliced *ABCB7* was determined by RT-PCR as described above.

*Panc 05.04 cell culture and cycloheximide treatment*

Pancreatic adenocarcinoma epithelial cell line, Panc 05.04 (ATCC) were cultured in RPMI medium 1640 (Sigma-Aldrich, Gillingham, UK) containing 15% fetal bovine serum and 1% Insulin-Transferrin-Selenium (ITS-G), at 37°C and 5% CO_2_. The cell line was identified to have a *SF3B1* (K700E) mutation by Sanger sequencing. Cells were treated with 100µg/ml cycloheximide for 4 hours and collected for RNA extraction. Total RNA was reverse transcribed using High capacity cDNA reverse transcription kit (Applied Biosystems). The expression of aberrantly spliced *ABCB7* was determined by RT-PCR as described above.

**Supplementary References**

1. Young MD, Wakefield MJ, Smyth GK, Oshlack A. Gene ontology analysis for RNA-seq: accounting for selection bias. *Genome Biol* 2010; **11**: R14.

2. Lawrence M, Huber W, Pages H, Aboyoun P, Carlson M, Gentleman R *et al*. Software for computing and annotating genomic ranges. *PLoS Comput Biol* 2013; **9**: e1003118.

3. Durinck S, Moreau Y, Kasprzyk A, Davis S, De Moor B, Brazma A *et al*. BioMart and Bioconductor: a powerful link between biological databases and microarray data analysis. *Bioinformatics* 2005; **21**: 3439-3440.

4. Durinck S, Spellman PT, Birney E, Huber W. Mapping identifiers for the integration of genomic datasets with the R/Bioconductor package biomaRt. *Nat Protoc* 2009; **4**: 1184-1191.

5. Crooks GE, Hon G, Chandonia JM, Brenner SE. WebLogo: a sequence logo generator. *Genome Res* 2004; **14**: 1188-1190.

6. Yeo G, Burge CB. Maximum entropy modeling of short sequence motifs with applications to RNA splicing signals. *J Comput Biol* 2004; **11**: 377-394.

7. Corvelo A, Hallegger M, Smith CW, Eyras E. Genome-wide association between branch point properties and alternative splicing. *PLoS Comput Biol* 2010; **6**: e1001016.

8. Fu Y, Sander JD, Reyon D, Cascio VM, Joung JK. Improving CRISPR-Cas nuclease specificity using truncated guide RNAs. *Nat Biotechnol* 2014; **32**: 279-284.

9. Desmet FO, Hamroun D, Lalande M, Collod-Beroud G, Claustres M, Beroud C. Human Splicing Finder: an online bioinformatics tool to predict splicing signals. *Nucleic Acids Res* 2009; **37**: e67.
