# Supplementary material for: Cryptic splicing events in the iron transporter ABCB7 and other key target genes in SF3B1-mutant myelodysplastic syndromes
Source: Leukemia. 2016 Jun 17;30(12):2322–31. doi: 10.1038/leu.2016.149 (PMC5029572; doi:10.1038/leu.2016.149)
Supplement: Supplementary Figures S1–S3 [file leu2016149x2.ppt]

## Slide 1
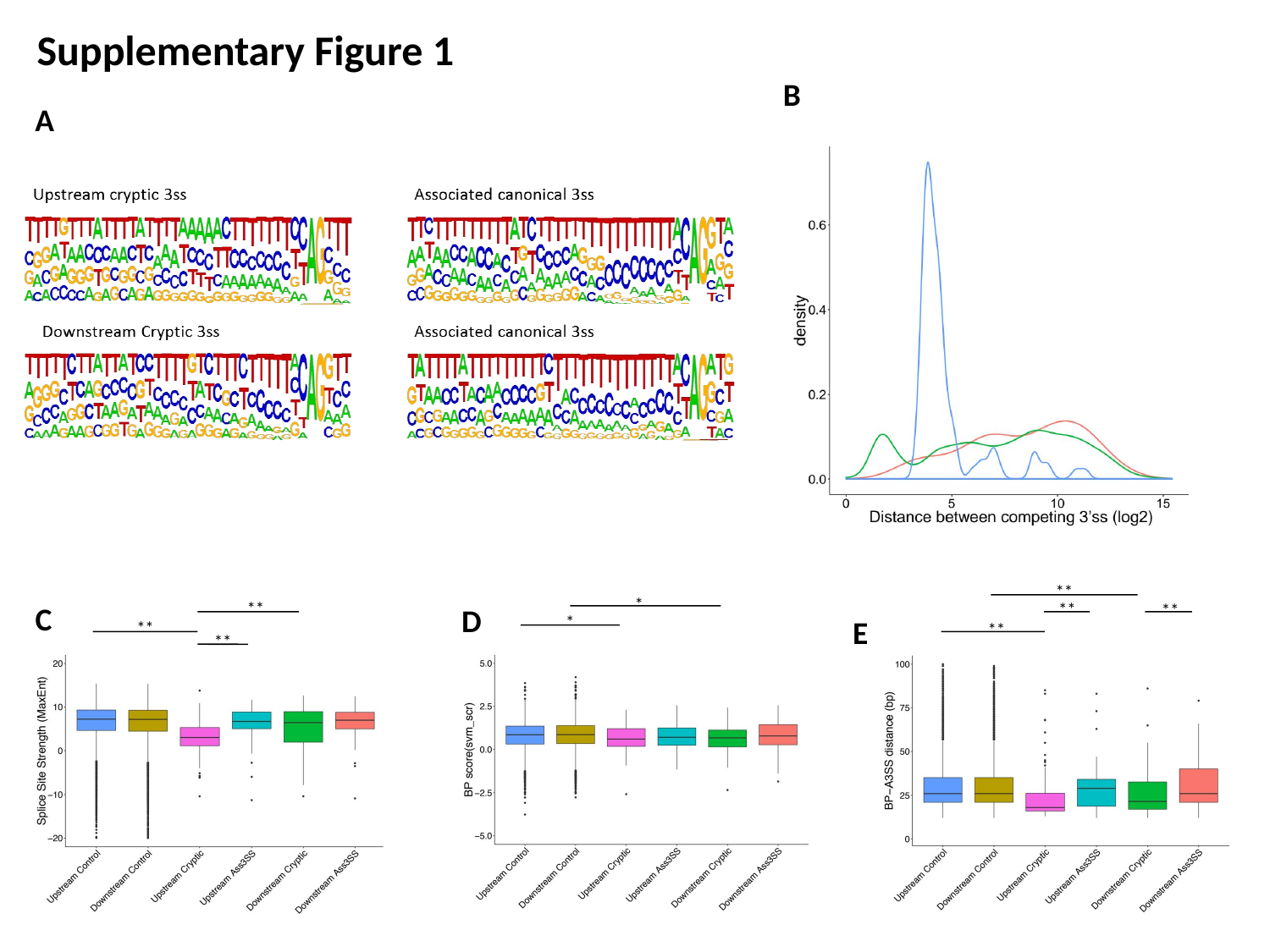

Supplementary Figure 1
B
A
**
*
**
**
**
C
D
*
E
**
**
**

## Slide 2
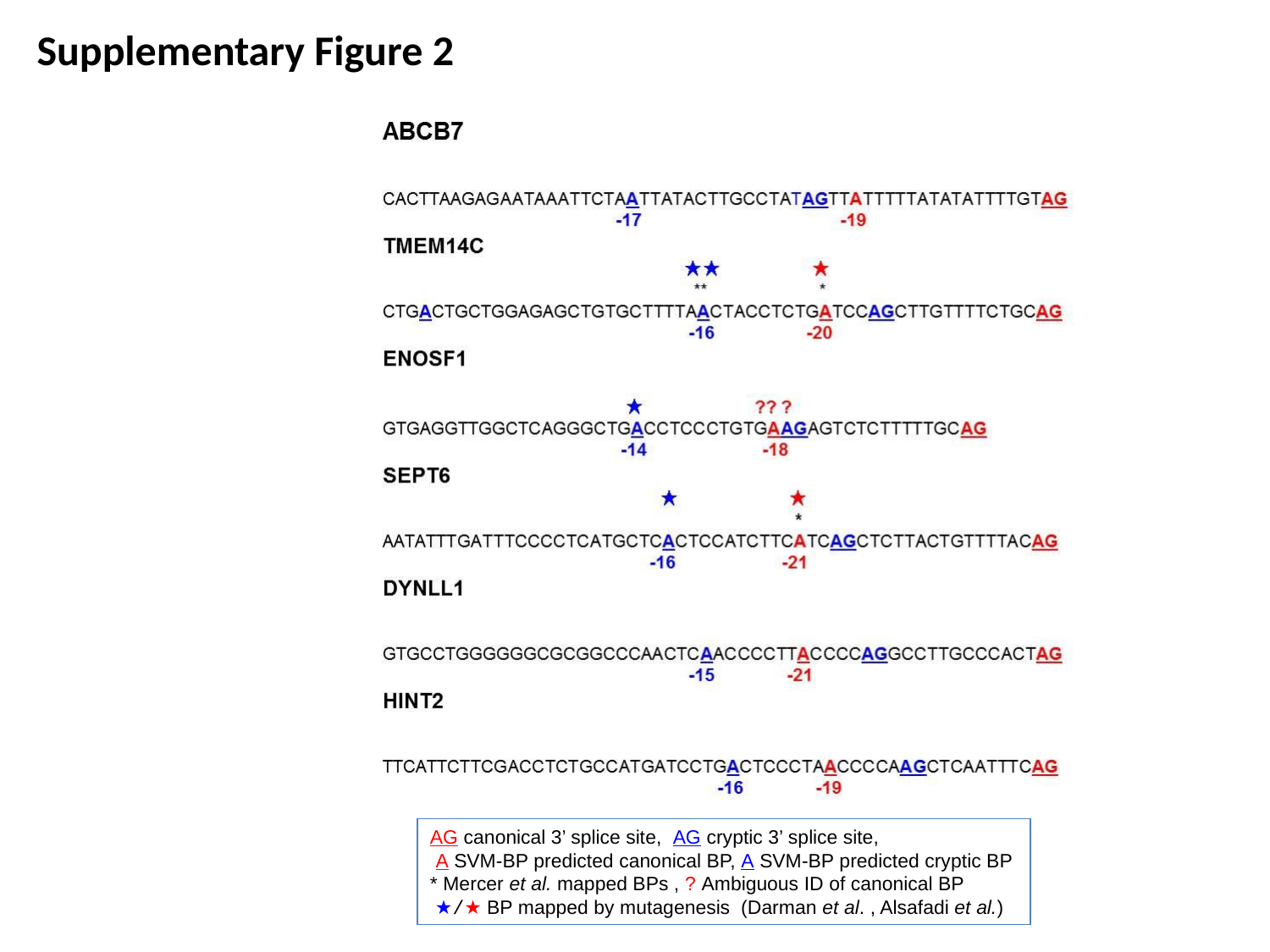

Supplementary Figure 2
AG canonical 3’ splice site, AG cryptic 3’ splice site,
 A SVM-BP predicted canonical BP, A SVM-BP predicted cryptic BP
* Mercer et al. mapped BPs , ? Ambiguous ID of canonical BP
 ★/★ BP mapped by mutagenesis (Darman et al. , Alsafadi et al.)

## Slide 3
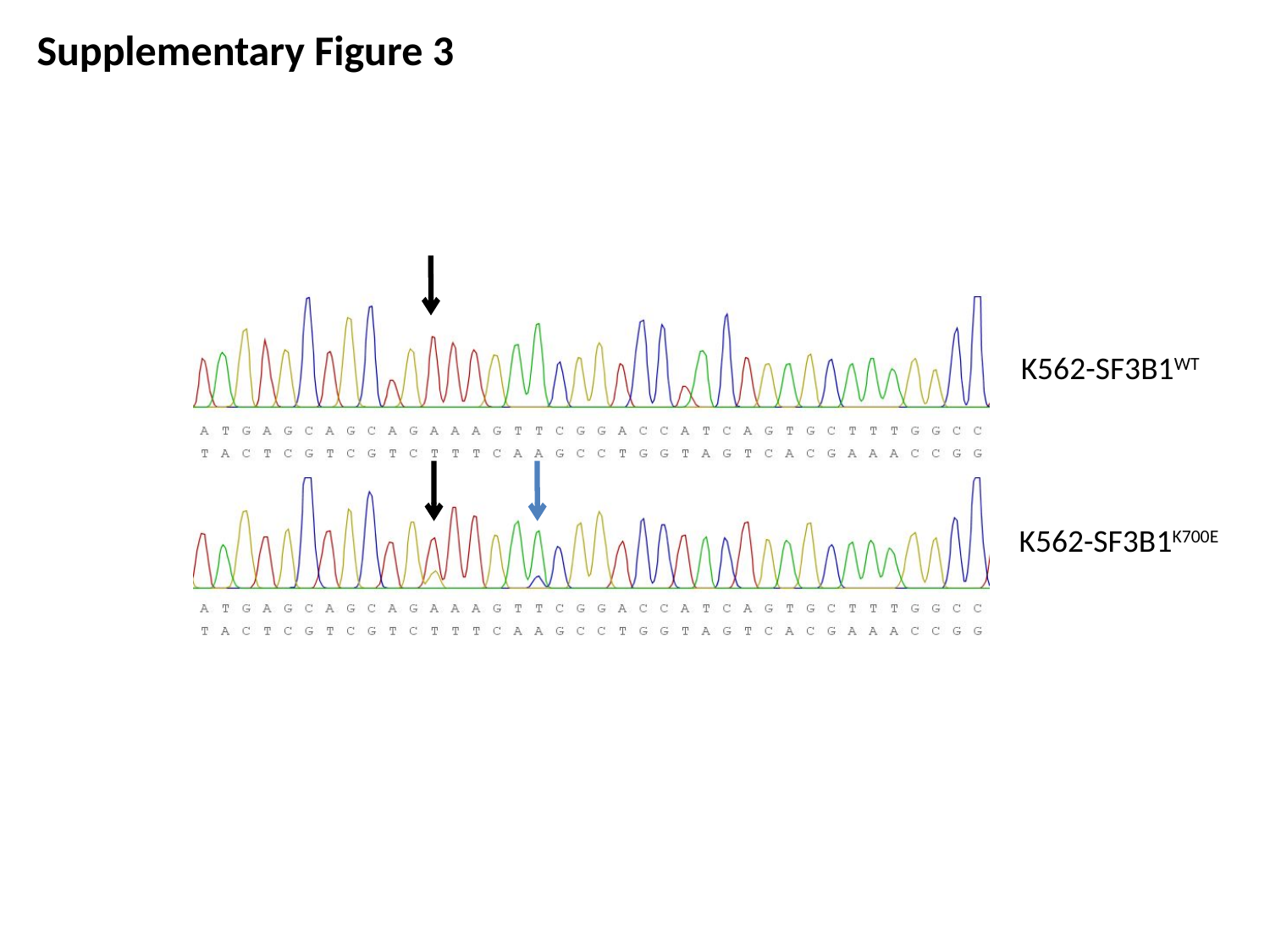

Supplementary Figure 3
K562-SF3B1WT
K562-SF3B1K700E
